# A Review of Randomized Controlled Trials for Vitiligo Therapies Published Between 2013 and 2023

**DOI:** 10.7759/cureus.112280

**Published:** 2026-07-08

**Authors:** Natalia Maverakis Ramirez, Zachary J Jaeger, Delphine J Lee

**Affiliations:** 1 Medicine, San Juan Bautista School of Medicine, Caguas, USA; 2 Dermatology, University of California San Diego, San Diego, USA; 3 Medicine, David Geffen School of Medicine at University of California Los Angeles (UCLA), Los Angeles, USA; 4 Medicine, The Lundquist Institute, Torrance, USA

**Keywords:** rct, repigmentation, treatments, vasi, vitiligo

## Abstract

Vitiligo is a depigmentation disorder whose treatment remains a serious challenge. While it is generally accepted that topicals and phototherapy are helpful for generalized symmetric disease, randomized controlled trials (RCTs) provide the best evidence for treatment. Our objective was to identify and summarize RCTs for vitiligo from 2013 to 2023. A systematic review registered with the International Prospective Register of Systematic Reviews (PROSPERO) was performed per PRISMA guidelines.

We searched CENTRAL, ClinicalTrials.gov, Embase, PubMed, and Web of Science for RCTs using keywords such as 'vitiligo' and/or 'treatment' or 'intervention.' A total of 652 studies underwent full-text review, and 151 studies met the inclusion criteria. We focused our study on RCTs using the vitiligo area scoring index (VASI) as the primary outcome measure, leading to 36 studies. We further narrowed our focus to studies that could be aggregated into the intervention categories: phototherapy and systemic combination therapy (n=10), topical and topical combination therapy (n=15), and systemic monotherapy (n=5).

Treated versus control subjects showed statistically improved VASI after the following interventions were added to phototherapy: oral psoralen, oral minipulsed prednisone, implanted afamelanotide, topical ethyl vanillate, topical bimatoprost, and oral vitamins A and E. Effective topical therapies were ruxolitinib, calcipotriol and betamethasone, tacrolimus and mometasone, and microdermabrasion and tacrolimus. None of the systemic monotherapies was superior to their corresponding comparator.

## Introduction and background

Vitiligo is a depigmentation disorder caused by autoimmune melanocyte destruction. Pathogenesis is regarded as multifactorial, with many genes and histocompatibility antigens associated with incidence. Vitiligo has also been linked to immunological changes, oxidative stress, morphological changes in the epithelium and upper dermis, environmental damage, and the possibility of neural etiology in some cases [1]. As pathogenic pathways for this disease may vary and successful complete repigmentation treatments are not well established, there exists a wide variety of approaches to therapy.

The United States has a vitiligo incidence rate of 22.6 per 100,000 person-years and a prevalence of 0.16% [2]. Worldwide prevalence is estimated to be between 0.1% and 8% [3]. Vitiligo can lead to psychosocial consequences due to difficulty finding effective treatment, maintenance of repigmentation, side effects, and social stigma experienced by patients. For example, in a descriptive study using Adelphi Real World data of 326 patients, physicians reported that patients experienced a detrimental impact in the following categories: everyday life (56%), emotional impact (47%), low self-esteem (44%), depression (28%), and anxiety (37%) [4]. Vitiligo impacts patients’ quality of life in several areas, such as social situations (30%), outdoor activities/sports (19%), professional life (16%), and intimacy (16%) [4], and is commonly comorbid with other autoimmune diseases, adding to the overall burden of disease. Therefore, while vitiligo is non-life-threatening, treatment and maintenance of repigmentation are a high priority.

Continued assessment of current and developing therapies is critical to ensure patients receive sound advice and evidence-based treatment plans. The last systematic review of all vitiligo treatments was published in 2015 and gathered data through 2013 [5]. Here, we present a systematic review aimed at summarizing advances in the field of vitiligo treatment over a decade, between 2013 and 2023.

A portion of this work was presented as a poster at the 2025 Society for Investigative Dermatology Annual Meeting.

## Review

Study design

**Figure 1:**
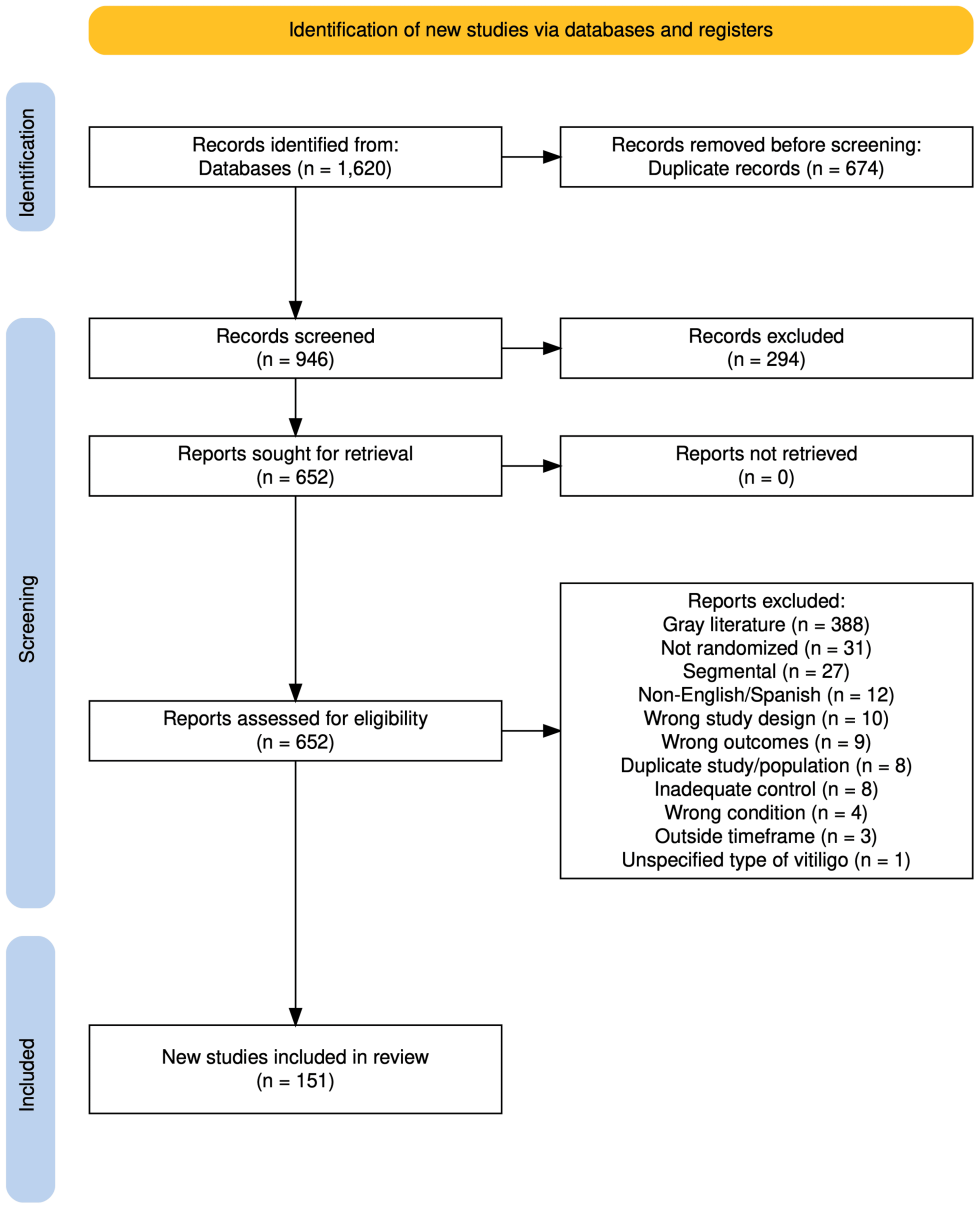
Systematic review flow diagram After exclusion of ineligible reports, 652 studies underwent full-text review, and 151 studies met the inclusion criteria. This diagram was created using the PRISMA flow diagram tool [6].

After removal of duplicates, 946 records were screened, 652 full-text articles were assessed, and 151 RCTs (Table 1) met the inclusion criteria for the systematic review. A PRISMA-compliant flow diagram was generated using the Shiny app by Haddaway et al. (Figure 1) [6]. This systematic review is registered in the PROSPERO database (CRD42023458292) and is compliant with PRISMA guidelines. 

**Table 1:** Number of clinical trials done in each intervention category from 2013 to 2023 Each RCT was categorized as described in the text. Combination therapies are denoted by a '+' between each category. The rows list intervention categories, along with references to the RCTs that investigated the intervention method. The 'Count' column shows all 151 RCTs included in the study [7-157]. RCT: Randomized controlled trial

Intervention category	Reference	Count
Excimer	[7]	1
Excimer + Intralesional	[8,9]	2
Excimer + Laser + Topical	[10]	1
Excimer + Phototherapy	[11]	1
Excimer + Surgical	[12-14]	3
Excimer + Systemic + Topical	[15]	1
Excimer + Topical	[16-24]	9
Intralesional	[25]	1
Intralesional + Phototherapy + Surgical	[26]	1
Intralesional + Laser	[27,28]	2
Intralesional + Laser + Phototherapy	[29]	1
Intralesional + Phototherapy	[30]	1
Intralesional + Surgical	[31,32]	2
Laser	[33]	1
Laser + Phototherapy	[34-37]	4
Laser + Phototherapy + Surgical	[38,39]	2
Laser + Phototherapy + Topical	[40-47]	8
Laser + Surgical	[48]	1
Laser + Systemic + Topical	[49,50]	2
Laser + Topical	[51-57]	7
Other	[58]	1
Phototherapy	[59-64]	6
Phototherapy + Intralesional	[65,66]	2
Phototherapy + Surgical	[67-74]	8
Phototherapy + Surgical + Systemic	[75]	1
Phototherapy + Surgical + Topical	[76]	1
Phototherapy + Systemic	[77-94]	18
Phototherapy + Topical	[95-110]	16
Surgical	[111-116]	6
Surgical + Topical	[117-128]	12
Systemic	[129-137]	9
Topical	[138-157]	20
Total included RCTs		151

Characteristics of the included studies

**Figure 2:**
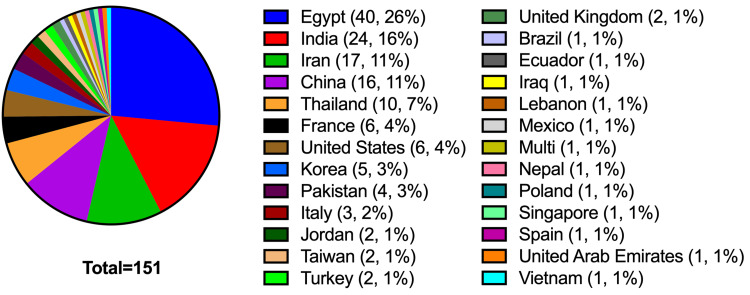
Countries that conducted RCTs on vitiligo from 2013 to 2023 The five countries publishing the most RCTs for vitiligo were: Egypt (40, 26%), India (24, 16%), Iran (17, 11%), China (16, 11%), and Thailand (10, 7%) [7-157]. This diagram was created using Prism 10 for macOS (GraphPad Software LLC, Boston, MA, USA). RCTs: Randomized controlled trials

The 151 studies collectively included a range of participant numbers (n=4 to 674 individuals per study), with ages spanning from three to 91 years [7-157]. The treatment durations varied between two weeks and 33 months. The top five countries producing the most RCTs of vitiligo between 2013 and 2023 were Egypt (40, 26%), India (24, 16%), Iran (17, 11%), China (16, 11%), and Thailand (10, 7%) (Figure 2).

Risk of bias assessment 

**Figure 3:**
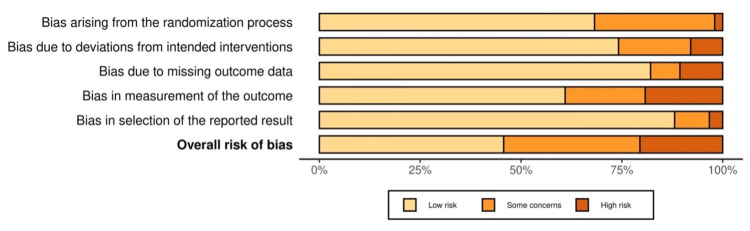
Overall risk of bias of all included RCTs (total n=151) Risk of bias of the RCTs (n=151) was assessed using the revised Cochrane risk of bias tool for randomized trials (RoB 2) [158]. The risk of bias plot was made using the robvis tool (R Package; The R Foundation for Statistical Computing, Vienna, AUT). Overall risk of bias demonstrated low risk in 45.8% of studies, some concerns in 33.7% of studies, and high risk in 20.5% of studies [7-157].

Overall risk of bias demonstrated low risk in ~45.8% of studies, some concerns in ~33.7% of studies, and high risk of bias in ~20.5% of studies (Figure 3) [7-157]. Efficacy of treatment was defined as a statistically significant p-value (p <0.05) versus control.

Individual therapies studied 

The therapies listed below are from RCTs that met full-text review criteria and were included in the final review. Interventions are categorized by light, surgical, systemic (including intramuscular injections and subcutaneous IVs), intralesional, topical, and other therapies. 

Light Therapies

Light therapies were classified by this systematic review as those involving phototherapy, excimer, or laser treatments. There were 60 RCTs investigating narrowband ultraviolet B (NB-UVB) radiation, six on ultraviolet A (UVA) radiation, 19 on excimer, and 20 on lasers. Existing literature supports that NB-UVB is more effective than UVA, and NB-UVB treatment for 12 months results in a greater chance of >75% repigmentation as opposed to shorter treatment durations [158]. Literature on excimer laser suggests that monotherapy is less effective than in combination with topical antioxidants [159]. Of the remaining lasers, such as carbon dioxide (CO₂), erbium-doped yttrium aluminium garnet (Er:YAG), and titanium:sapphire, only CO₂ lasers have been reviewed via meta-analysis and have shown promise as adjunct therapy [160]. Excimer therapies in this systematic review included 308-nm excimer lamp [10,14,18,19,64], excimer laser [8,9,11,13,15,17,20-22,24,50,53], and cyclic excimer [24]. Excimer lasers emit monochromatic light at 308 nm [161]. In contrast to NB-UVB, which uses several lamps and exposes all the patient’s skin, the excimer laser allows targeted treatment of vitiligo-affected areas. The CO₂ laser treatment exists in both short-pulsed CO₂ and fractional CO₂. Short-pulsed CO₂ treatment uses a 10,600-nm wavelength to ablate tissue quickly, with the hopes of minimizing skin damage and scarring. Fractional CO₂ laser, in contrast, uses microbeams in a fractionated pattern and is postulated to yield quicker recovery than short-pulsed emission (Table 2) [51,162-164].

**Table 2:** Studies reporting the efficacy of topical and topical combination treatments in patients with vitiligo The rows list studies that included VASI as an outcome measure and investigated topical or topical combination therapies for non-segmental vitiligo [51,95,96,98,99,117-119,138-144]. The intervention column lists the primary treatment being tested first, followed by control(s). Duration of treatment reports the endpoint of the trial in weeks, months, or years. When available, changes in VASI and percentage changes in VASI were either reported directly from the RCT or calculated from available pre- and post-treatment VASI values. Errors are displayed as mean +/- standard deviation (SD). NI: Not included, VASI: Vtiligo area scoring index, ANOVA: Analysis of variance​​​​​​​, ^†^Denotes independent t-test, ^‡^Denotes Mann-Whitney U test, ^§^Denotes Chi-Square test, *Denotes paired t-test, ^±^Denotes logistic regression, ^¶^Denotes Friedman test, ^„^Denotes repeated-measures ANOVA with Bonferroni correction, ˆDenotes repeated-measures ANOVA, ^‹^Denotes Wilcoxon matched-pairs signed-rank test

Authors	Intervention	n	Body site	Age, mean (range), y	Duration	Percentage change in VASI, treatment group	Statistic: Controls vs. baseline	Statistic: Treatment vs. baseline	Statistic: Treatment vs. controls	Statistic across 3+ treatments
Abd-Elazim et al., 2020 [117]	1: Microdermabrasion + tacrolimus, 2: Tacrolimus	35	NI	36 (8-59)	3 months	−31.9 (10.94)	NI	NI	t<.001^†^	N/A
AbuZeid et al., 2020 [51]	1: Fractional CO2 laser + tacrolimus, 2: Tacrolimus	27	All sites	31.96	3 months	-10.05	t=0.24^†^	t=0.46^ †^	NI	N/A
Asilian et al., 2021 [118]	1: Dermabrasion + 5-FU, 2: Suction blister	36	All sites	31.6 (13-65)	3 months	NI	NI	NI	NI	N/A
El-Magid et al., 2023 [119]	1: Microneedling + latanoprost, 2: Latanoprost	72	Acrofacial	(18-68)	3 months	-10	U=0.392^‡^	U=0.003^‡^	U=0.692^‡^	N/A
El-Zawahry et al., 2019 [95]	1: Topical PUVA, 2: UVA1	20	NI	NI	3 months	NI	U=0.109^‡^	U=0.102^‡^	NI	N/A
Jalalmanesh et al., 2022 [138]	1: Turmeric, 2: Placebo cream	30	All sites	NI	4 months	NI	NI	NI	X^2^<.001^§^	N/A
Kalafi et al., 2014 [96]	1: NB-UVB + tetracycline, 2: NB-UVB + placebo	30	All sites	(11-66)	3 months	-3.88	t=0.026^*^	t=0.026^*^	t=0.566^*^	N/A
Namazi et al., 2015 [98]	1: Ethyl vanillate + NB-UVB, 2: Eucerin + NB-UVB	30	All sites	(22-60)	3 months	-7.36	t=0.066^*^	t=0.002^*^	t=0.005^*^	N/A
Rosmarin et al., 2020 [139]	1: 1·5% ruxolitinib cream twice daily, 2: Vehicle cream	157	Face	48.3 (18-73)	24 weeks	NI	NI	NI	p=0.0001^±^	NI
Rosmarin et al.,2022 [140]	1: 1·5% ruxolitinib cream twice daily, 2: Vehicle cream	674	Face	40.5/38.4	24 weeks	NI	NI	NI	p<0.001^±^/p<0.001^±^	NI
Saleh et al., 2021 [141]	1: Tacrolimus, 2: Hydrocortisone acetate	63	All sites	(3-75)	6 months	-33.33	NI	X^2^=0.03^¶^	X^2^=0.862^¶^	N/A
Silpa-Archa et al., 2023 [99]	1: Bimatoprost + NB-UVB, 2: Bimatoprost, 3: Saline solution	13	All sites	(14-53)	6 months	NI	NI	NI	1: F<.001,^„ ^2: F<.001^„^	F=0.001^„^
Venkatesan et al., 2021 [142]	1: Tacrolimus + mometasone furoate, 2: Tacrolimus	50	All sites	NI	12 months	NI	NI	NI	t<0.05^†^	N/A
Zahoor et al., 2017 [143]	1: Betamethasone dipropionate + calcipotriol, 2: Calcipotriol, 3: Betamethasone dipropionate	159	All sites	NI	3 months	NI	NI	NI	2: F=0.006ˆ, 3: F=0.134ˆ	F=0.384ˆ
Zhai et al., 2020 [144]	1: Cold atmospheric plasma-activated hydrogel, 2: Vehicle hydrogel, 3: No treatment	20	NI	36 (12-68)	2-28 weeks	NI	2: t=0.258^†^, 3: t=0.778^†^	Z=0.0012^‹^	NI	NI

The Er:YAG laser's absorption coefficient is 12,800/cm, and has been shown to be 12 to 18 times more effective than the CO2 laser [165]. This ablative technique was first introduced by Kaufmann et al. in 1998 as an in vitro method to graft melanocytes to laser-ablated lesions in vitiligo [166]. The titanium:sapphire laser is a solid-state laser developed to emit light at 311 nm to mitigate adverse effects common at the 308 nm range, such as burning [167]. Broadband UVA (BB-UVA), or UVA, is light with a 320-400 nm wavelength and can affect the dermis [168]. The UVA1 is a 340-400 nm wavelength light. Longer wavelengths of light are thought to penetrate deeper into the dermis; thus, the basal layer is damaged to a greater degree than UVA alone [169]. It is hypothesized that this treatment method induces the production of reactive oxygen species and cyclobutane pyrimidine dimers. This consequently disrupts oxidative phosphorylation and generates DNA damage, thus inhibiting immune response and allowing melanocytes to proliferate [169]. Psoralen + UVA (PUVA) therapy, first developed in 1974 for psoriasis, is a combination of psoralen and UVA therapy (320-400 nm) [170]. Psoralen is a plant byproduct that photosensitizes skin, thereby allowing the patient to become more susceptible to UVA treatment. Psoralen is administered orally or cutaneously before UVA or NB-UVB therapy (Tables 3-4) [77,78,80-86,94,129-133]. 

**Table 3:** Studies reporting the efficacy of phototherapy and systemic combination treatments in patients with vitiligo The rows list studies that included VASI as an outcome measure and investigated phototherapy and systemic combination therapies for non-segmental vitiligo [77,78,80-86,94]. The intervention column lists the primary treatment being tested first, followed by control(s). Duration of treatment reports the endpoint of the trial in weeks, months, or years. When available, changes in VASI and percentage changes in VASI were either reported directly from the RCT or calculated from available pre- and post-treatment VASI values. Errors are displayed as mean +/- standard deviation (SD). NB-UVB: Narrowband ultraviolet B, UVB: Ultraviolet B, OMP: Oral mini-pulse, NI: Not included, VASI: Vitiligo area scoring index, ANCOVA: Analysis of covariance, ^‹^Denotes Wilcoxon matched-pairs signed-rank test, ^†^Denotes independent t-test, ^+^Denotes Kruskal–Wallis test, ˜Denotes unspecified statistical test, ^•^Denotes ANCOVA, ^‡^Denotes Mann-Whitney U test, ˆDenotes repeated-measures ANOVA

Author	Intervention	n	Body site	Age, mean (range), y	Duration	Percentage reduction in VASI, tx group	Statistic: Controls vs. baseline	Statistic: Treatment vs. baseline	Statistic: Treatment vs. controls	Statistic across 3+ treatments
Bansal et al., 2013 [77]	1: Psoralen + NB-UVB, 2: NB-UVB	45	All sites	(13-70)	5 months	29.20%	Z<0.001^‹^	Z<0.001^‹^	t=0.043^†^	N/A
Charoenpongpun et al., 2022 [78]	1: Oral minocycline + NB-UVB, 2: Oral placebo + NB-UVB	14	All sites	33.4	3 months	28.87%	NI	NI	t=0.886^†^	N/A
El Mofty et al., 2016 [80]	1: NB-UVB + prednisone OMP, 2: Prednisone oral mini-pulse (OMP) 3: NB-UVB	45	NI	NI	3 months	60.60%	3: Z=0.002^‹^	Z=0.001^‹^	NI	t=0.041^+^
Garza-Davila et al., 2023 [81]	1: Vitamin D + NB-UVB, 2: Oral placebo + NB-UVB	15	NI	41.5	6 months	63.01%	p=0.18 ^˜^	p=0.003 ^˜^	p=0.465 ^˜^	N/A
Jowkar et al., 2020 [82]	1: NB-UVB + silybum marianum, 2: NB-UVB + placebo	40	All sites	34.4 (15-67)	3 months	23.14%	Z=0.003^‹^	Z=0.001^‹^	Z=0.202^‹^	N/A
Khemis et al., 2020 [83]	1: NB-UVB + apremilast, 2: NB-UVB + oral placebo	80	All sites	NI	6 months	22.48%	F<0.0001^•^	F=0.011^•^	F=0.18^•^	N/A
Lim et al., 2015 [84]	1: Afamelanotide implant + NB-UVB, 2: NB-UVB	55	All sites	18-79	5 months	49%	U<.001^‡^	U<.001^‡^	U<.05^‡^	N/A
Nguyen et al., 2018 [94]	1: Atorvastatin + NB-UVB, 2: NB-UVB	30	NI	45 (27-63)	6 months	NI	F=0.04^•^	F=0.005^•^	F=0.49^•^	N/A
Nowowiejska et al., 2023 [85]	1: Vitamin A+E + UVB 311nm, 2: Vitamin A+E, 3: UVB 311nm	66	NI	41.5	4 months	38.44%	2: t=0.018^†^3: t=0.001^†^	t<0.001^†^	t<0.001^†^	t<0.001^+^
Zabolinejad et al., 2020 [86]	1: Psoralen + NB-UVB, 2: NB-UVB	40	All sites	33.9	20 weeks	46.39%	F=0.011 ˆ	F=0.008 ˆ	F=0.026 ˆ	N/A

**Table 4:** Studies reporting the efficacy of systemic treatments in patients with vitiligo The rows list studies that included VASI as an outcome measure and investigated systemic monotherapies for non-segmental vitiligo [129-133]. The intervention column lists the primary treatment that was tested first, followed by the control(s). Duration of treatment reports the endpoint of the trial in weeks, months, or years. When available, changes in VASI and percentage changes in VASI were either reported directly from the RCT or calculated from available pre- and post-treatment VASI values. Errors are displayed as mean +/- standard deviation (SD). NI: Not included, VASI: Vitiligo area scoring index, ^‡^Denotes Mann-Whitney U test, ^†^Denotes independent t-test, ^‹^Denotes Wilcoxon matched-pairs signed-rank test, ˜Denotes unspecified statistical test

Author	Intervention	n	Body site	Age, mean (range), y	Duration	Percentage reduction in VASI, treatment group	Statistic: Controls vs. baseline	Statistic: Treatment vs. baseline	Statistic: Treatment vs. controls
Abu-Raghif et al. 2013 [129]	1: Ginkgo biloba, 2: Oral placebo	50	NI	(18-58)	8 weeks.	-3.89	NI	NI	U=0.126^‡^
Mehta et al. 2021 [130]	1: Cyclosporine, 2: Dexamethasone OMP	50	NI	NI	4 months	-7.43	NI	NI	t=0.88^†^
Singh et al. 2014 [131]	1: Minocycline, 2: Dexamethasone OMP	50	NI	NI	6 months	-11.11	Z=0.05^‹^	Z=0.06^‹^	Z=0.11^‹^
Singh et al. 2015 [132]	1: Methotrexate, 2: Dexamethasone OMP	52	NI	NI	6 months	3.07	NI	NI	NI
Vanderweil et al. 2017 [133]	1: Simvastatin, 2: Oral placebo	15	NI	NI	6 months	26	NI	NI	p=0.094^˜^

The NB-UVB (310-315 nm) was first introduced in 1997 as a vitiligo treatment and has since become one of the most widely accepted treatments for vitiligo. It stimulates the melanocytes around hair follicles to proliferate, creating a cascade of melanocyte migration. While its mechanism of action is not completely understood, some postulate that protein kinase A, protein kinase C, and mitogen-activated protein kinase (MAPK) all contribute to melanocyte differentiation [171]. BIOSKIN® (NB-UVB microphototherapy) emits light at 311 nm and is described as a 'cold light,' or one that produces less heat as a result of fiber optic cables being used for the transmission of light as opposed to glass [172,173].

Heliotherapy, or natural sunlight, was one of the original treatments for vitiligo, with first mentions dating back to the Ebers Papyrus in 1500 BC [174]. The PUVASOL therapy administers psoralen in a similar manner to traditional PUVA therapy. However, sunlight is used as the source of UVA light. 

Surgical Therapies

Surgical interventions for vitiligo are heterogeneous and numerous, and existing literature suggests that surgical monotherapy for vitiligo results in repigmentation. For example, a meta-analysis of 117 studies found that after one round of surgical treatment, 52.69% of included studies resulted in greater than or equal to 90% repigmentation [175]. 

Microneedling is a minimally invasive technique in which a dermaroller is applied to the skin to create micropunctures. These wounds activate the healing cascade, stimulating fibroblast growth factor, TGF-β, VEGF, PDGF, EGF, and metalloproteinases. Typically, this is an adjuvant therapy to be used in combination with topical treatments, allowing for improved delivery of topical drugs beyond the stratum corneum (Table 2) [176]. Acupuncture was classified by this review as another type of microneedling. 

Electrocautery needling is a method of inducing heat and mechanical energy to the lesional skin to promote repigmentation. It is thought to be inspired by fire needle therapy, common in traditional Chinese medicine, in which acupuncture needles are heated and quickly inserted into the skin [177].

In punch and split skin grafting, donor or pigmented skin is transplanted onto vitiligo-affected skin. The diameter of the punch graft ranges from 1 mm to 2 mm in thickness. Split-thickness grafting is accomplished by using a dermatome blade to obtain a thin skin graft for transplant onto a vitiligo-affected area [178]. Melanocyte transplantation via these methods prompts the proliferation of new melanocytes, which spread to non-grafted zones, a phenomenon described as 'satellite repigmentation' [179].

Suction blister epidermal grafting uses a syringe to create blisters on the pigmented donor site. The resulting blister only contains the epidermis and is then transferred onto recipient patches of skin [180]. This method introduces melanocytes into the affected areas, encouraging repigmentation. This technique is often used for stable vitiligo and is considered when other treatments have not been successful. Cryoblister creates a controlled blister using liquid nitrogen. The epidermis, containing healthy melanocytes, is then transplanted onto lesional skin. 

In smash skin grafting, non-lesional skin is harvested and kept hydrated with minimal amounts of saline. It is then minced until a homogeneous mixture is formed and placed on top of the lesional skin and covered with gauze [73].

Cultured melanocyte transplant is achieved by taking a biopsy of pigmented skin and culturing the melanocytes before being transplanted onto a recipient site. However, this method is time-consuming; thus, noncultured epidermal suspension is favored. In a noncultured epidermal suspension, a pigmented biopsy is taken, put into suspension, and then directly applied as a cell suspension to a prepped recipient site [181].

Follicular transplantation can also be used as a means of repigmentation, as the bulge and hair germ region of telogen phase follicles contains melanocyte stem cells (McSC) [182]. These stem cells can be deposited into vitiliginous areas to create new melanocytes. This method was first explored in 1998 and produced perifollicular repigmentation [183]. Introducing hair follicle stem cells to lesional skin can be achieved by transplantation via single hair grafting and cultured or uncultured cell suspension. 

Platelet-rich plasma is a portion of plasma that has a high concentration of platelets compared to normal blood. Its effects can be categorized into anabolic effects and anti-inflammatory effects [184]. It is known to increase the release of growth factors, which stimulate fibroblasts and keratinocytes, while also providing anti-inflammatory effects through the inhibition of cytokines. 

Dermabrasion is a method of skin resurfacing that erodes the epidermis and superficial dermis. It was first employed as a treatment for vitiligo in 1985 by manual sandpaper abrasion or an electric rotary device to topically administer fluorouracil [185]. 

Systemic Therapies

Corticosteroids are commonly used to treat vitiligo by broadly suppressing the immune response, thus halting the destruction of melanocytes. When used systemically, the oral mini-pulse (OMP) method of delivery is preferred to minimize systemic side effects, which involves dosing on two consecutive days per week for approximately three months [186]. A review conducted in 2021 stated that more research on OMP must be conducted to conclude clinical utility [187]. Most of the OMP corticosteroid therapies analyzed in this review served as a head-to-head comparator against another systemic immunosuppressant for stabilizing active vitiligo, and they generally worked well for arresting progression of disease [130-132,134,135,137]. The combination of OMP corticosteroids and NB-UVB versus placebo with NB-UVB demonstrated significant improvement (p = 0.041) (Table 3) [80] or no added benefit [87]. 

Apremilast is a phosphodiesterase-4 (PDE4) inhibitor that increases cyclic adenosine monophosphate (cAMP) levels. This second messenger is employed in several pathways, decreasing IFN-γ, CXCL9, CXCL10, and IL-2, 12, 17, and 23, while upregulating IL-10, leading to decreased immune destruction of melanocytes. Apremilast can also induce repigmentation in vitiligo via activation of melanocyte-inducing transcription factor (MITF) by cAMP [188]. The NB-UVB combined with apremilast provided no additional benefit versus NB-UVB with placebo (Table 3) [83].

Minocycline is an antibiotic that was first considered as a potential treatment for vitiligo in 2008, when high levels of hydrogen peroxide were reported in plasma and skin lesions of vitiligo patients. It has antioxidant properties and was found to protect murine melanocytes against hydrogen peroxide-induced cell death via Janus kinase (JAK) and p38 mitogen-activated protein kinase pathways in vitro [189]. Minocycline combined with NB-UVB provided no additional benefit compared to NB-UVB and placebo, and minocycline did not perform better than NB-UVB in a head-to-head study (Table 3) [78,79]. Lastly, minocycline (versus OMP dexamethasone) failed to demonstrate improved VASI (Table 4) [131].

The JAK inhibitors are broadly immunosuppressive small-molecule inhibitors of receptor tyrosine kinases. The concept of inhibiting JAK/signal transduction and activation of transcription (STAT) as a treatment for vitiligo stems from the idea that melanocyte-specific CD8+ T-cells, as well as the cytokines IFN-γ, IL-2, and IL-15, contribute to melanocyte destruction [190]. The CD8+ T-cells produce IFN-γ, which activates the JAK/STAT1 pathway. The IFN-γ induces the T-cell chemokine receptor CXCR3 and its ligands, CXCL9/10/11, which are significantly elevated in vitiligo lesions [191]. A recent meta-analysis also found CCL5, CXCL8, CXCL12, and CXCL16 to be elevated; these could serve as future therapeutic targets in vitiligo treatment [192]. By inhibiting the JAK/STAT pathway, the release of MMP-9, which makes E-cadherin soluble and leads to melanocyte detachment and apoptosis via the chemotaxis of CD8+ lymphocytes, is decreased. Since IFN-γ activates the JAK/STAT pathway, inhibition of the pathway can block the production of CCL9 and CCL10, which are responsible for recruiting melanocyte-targeting T-cells and recruiting T-cells in the skin, thus promoting and prolonging depigmentation. The selective JAK3 inhibitor ritlecitinib demonstrated significant improvement in facial VASI75 for 50 mg daily with or without a loading dose period (p < 0.001) and for 30 mg daily (p = 0.01), with a trend toward significance for total VASI at 50 mg daily with a 100 mg daily four-week loading dose (Table 2) [136].

The HMG-CoA reductase inhibitors (statins) exhibit immunomodulatory effects in patients with acute coronary syndrome, notably increased IL-10 and TGF-β levels and decreased IFN-γ levels [193]. Therefore, they were also tested for vitiligo. The addition of atorvastatin to phototherapy provided no additional benefit over phototherapy alone (Table 3) [94]. Simvastatin showed no effect in a placebo-controlled trial (Table 4) [133].

Methotrexate is an antimetabolite that inhibits dihydrofolate reductase, an enzyme essential for DNA and RNA synthesis. It has anti-inflammatory properties, specifically increasing IL-10 and IL-4, while decreasing IL-1, IL-2, TNF-α, and IFN-β [194]. The combination of methotrexate 15 mg weekly and dexamethasone OMP significantly improved repigmentation versus either agent alone in one trial (p < 0.001 and p < 0.05) [135]. Another study demonstrated the effectiveness of methotrexate 10 mg weekly in halting the progression of active vitiligo, though the results were not superior to the dexamethasone OMP arm [105].

Azathioprine is a purine analog that is converted to 6-mercaptopurine and interrupts DNA synthesis to exert immunosuppressive effects via reduced proliferation of B and T lymphocytes. A trial for rapidly progressing vitiligo showed non-statistically significant arrest of disease for azathioprine 50 mg twice daily compared to betamethasone OMP, and overall repigmentation was minimal for most patients on azathioprine [137].

Cyclosporine is a calcineurin inhibitor. Inhibition of IL-2 production decreases activation of T-lymphocytes and downregulates TNF-α and IFN-γ, inflammatory mediators contributing to autoimmune destruction of melanocytes [195]. Cyclosporine has also been shown to aid in DNA repair via DNA-dependent protein kinase. Network pharmacology and validation via molecular docking have identified overlapping gene targets for vitiligo and cyclosporine, notably CUL1, HSP70, and SIRT7 [196]. Cyclosporine 3 mg/kg/day was as efficacious in stabilizing rapidly progressing vitiligo as the comparator group receiving dexamethasone OMP; repigmentation outcomes were not significant between groups (Table 4) [130].

Mycophenolate mofetil is an immunosuppressive drug that is converted to mycophenolic acid (MPA) in the body. The MPA inhibits inosine monophosphate dehydrogenase, which is needed for B and T lymphocyte proliferation [197]. In vivo studies have shown that MPA also impairs the maturation and function of dendritic cells in vivo, which would impair their ability to present antigen to T cells, thus decreasing inflammation [198]. Neither mycophenolate mofetil nor the comparator group, dexamethasone OMP (2.5 mg, two successive days a week for 180 days), produced significant changes in VASI from baseline when used as monotherapy, though it is important to mention that this trial analyzed a relatively small number of patients in the treatment and control groups (n=18 and 20, respectively) [134]. However, mycophenolate mofetil and dexamethasone OMP were able to stabilize disease progression, as exemplified by statistically significant differences in vitiligo disease activity (VIDA) score, and mycophenolate mofetil resulted in a statistically significant VIDA in comparison to controls at the 270-day follow-up (p = 0.03).

Afamelanotide is a synthetic α-melanocyte-stimulating hormone (α-MSH) analog first introduced in 2009 to decrease cutaneous photosensitivity in erythropoietic protoporphyria patients [199,200]. It stimulates melanin production by binding to melanocortin-1 receptors and stimulating differentiation of melanoblasts. Afamelanotide implant in combination with NB-UVB showed statistically improved VASI in comparison to NB-UVB monotherapy (p < 0.05) (Table 3) [84]. 

Oral vitamins A and E are potent antioxidants thought to address vitiligo through the reduction of oxidative stress. Triple combination therapy of NB-UVB, vitamin A, and E resulted in statistically improved VASI and was superior to both NB-UVB monotherapy and vitamin A and E combination therapy (p < 0.001) (Table 3) [85]. Vitamin D3 is considered to have immunomodulatory properties. A meta-analysis showed that in 1840 patients with vitiligo, circulatory levels of vitamin D were lower in vitiligo patients compared to controls (p < 0.00001) [201]. Vitamin D and NB-UVB combination therapy provided no additional benefit compared to NB-UVB control (Table 3) [81].

*Polypodium leucotomos* is an antioxidant fern native to Central and South America, commonly found in the canopies of rainforests. It has been shown to inhibit oxidative damage, protect mitochondrial DNA, and prevent the formation of cyclobutane pyrimidine dimers [202,203].* Polypodium leucotomos *also blocks AP-1, iNOS, NF-KappaB, and TNF-α [204]. One study found that the addition of *Polypodium leucotomos* to NB-UVB was superior to NB-UVB alone after three months of follow-up, though only for the head and neck region (p < 0.001) [91]. Another trial similarly concluded that the addition of *Polypodium leucotomos* to NB-UVB resulted in an increase in repigmentation over NB-UVB and placebo, though statistical analysis was not conducted [92]. 

Silybum marianum was first used for the treatment of jaundice and is thought to be hepatoprotective. It has been considered a treatment of vitiligo due to its inhibition of myeloperoxidase in stimulated neutrophils [82,205,206]. Silybum marianum and NB-UVB combination therapy provided no additional benefit compared to NB-UVB control (Table 3) [82]. 

Alpha-lipoic acid (ALA) is an antioxidant that binds free radicals, one of the known factors involved in vitiligo pathogenesis [207]. The addition of ALA to intramuscular betamethasone and NB-UVB was effective in reducing vitiligo lesions versus placebo with intramuscular betamethasone and NB-UVB (p = 0.036) three months post-treatment, though the difference between groups was no longer significant at six months [89]. In another study, the addition of ALA to NB-UVB was not significant in comparison to NB-UVB control [93]. 

Glycyrrhizin has been shown in murine models of inflammation to attenuate immune responses by regulating Th1/Th2 balance [208] and is commonly used in Chinese medicine as an anticancer agent [209]. Addition of glycyrrhizin to NB-UVB was superior to NB-UVB alone (p < 0.05) [90]. It has also been used in combination with a CO₂ laser and triamcinolone, though repigmentation was not quantified [49]. *Ginkgo biloba* has been shown to decrease oxidative stress induced by hydrogen peroxide and aid in melanogenesis via the flavonoids baicalein, hesperetin, and naringenin [210,211]. The *Ginkgo biloba *RCT included in this study did not significantly reduce VASI (Table 4) [129]. 

Intralesional Therapies

Fluorouracil (5-FU) is a pyrimidine analog that inhibits thymidylate synthase, thus disrupting DNA synthesis in rapidly dividing cells such as keratinocytes, which have been implicated in the pathophysiology of vitiligo [212]. By removing dysfunctional keratinocytes, 5-FU allows for new keratinocytes to differentiate and aid in repigmentation. Other studies have found that 5-FU activates the CXCL12/CXCR4 axis, thereby influencing melanocyte migration and inducing pigmentation [213]. The 5-FU was administered once intralesionally, and monotherapy resulted in a significant decrease in the size of the vitiligo lesion versus triamcinolone acetonide control (p = 0.002) [25].

Prostaglandins such as PGE2 have a role in melanogenesis and melanocyte maturation. The PGF2α-analog latanoprost eyedrops are commonly known to cause hyperpigmentation of the iris and periocular skin; thus, it was hypothesized that they may be effective in treating vitiligo lesions. An RCT comparing intralesional PGE2, PGF2α, and saline placebo alongside NB-UVB in each group demonstrated significantly greater repigmentation rates in the prostaglandin-treated arms (p < 0.001) [30].

Carboxytherapy involves the intralesional injection of sterile purified CO₂ gas for the treatment of conditions including lipodystrophies, alopecia, and pigmentary disorders [214,215]. The formation of carbonic acid decreases the local pH and results in microvascular vasodilation and local growth factors. The only RCT investigating this treatment modality for vitiligo compared NB-UVB with and without carboxytherapy and showed increased proportions of repigmentation with combination therapy compared to NB-UVB control (p = 0.001) [66].

Topical Therapies

Calcineurin inhibitors were postulated as a treatment for vitiligo as early as 2002, when Smith et al. published a case report using tacrolimus as a treatment for vitiligo. The broad immunosuppressive properties of calcineurin inhibitors through blockade of IL-2 transcription and lymphokine production halt immune-driven destruction of melanocytes [216]. Tacrolimus was studied in 25 RCTs [10,17,21,22,24,50,51,53,56,101,104,107,110,117,120-122,141,142,145,150,153,155-157] and pimecrolimus in three RCTs [22,123,126]. The RCTs included in this review used topical calcineurin inhibitors as a control group, component of combination therapy, or primary comparator. When studied as a monotherapy, 0.03% tacrolimus ointment was superior to hydrocortisone ointment control (p < 0.001) (Table 2) [141]. 

Camouflage with cosmetics is another management option, as social acceptability remains an important consideration for individuals living with vitiligo. As such, in addition to directly treating the disease pathology causing depigmentation, it is beneficial to employ camouflage methods to improve quality of life. Tan or brown pigments can effectively disguise visible depigmented skin. In one study, formulations of traditional Iranian Sabgh and a marketed camouflage, Exuviance®, both significantly improved Dermatology Life Quality Index (DLQI), but neither was superior to the other [148].

Important considerations in topical corticosteroid use include the vehicle for delivery as well as corticosteroid potency. Ointments are the most potent due to their occlusive nature, followed by creams, lotions, gels, and foams. The location of the vitiligo should be considered when selecting a vehicle, as lotions, gels, and foams work well in hair-bearing regions [217]. The vasoconstrictor assay is the gold standard for potency determination. The United States classifies steroids from class I (super potent) to VII (least potent) [218]. Class I-III potencies are typically used for vitiligo as a first-line treatment in combination with UVB, as they provide some efficacy but have limitations with regard to side effects with generalized or frequent application [219]. The topical steroids betamethasone, clobetasol, mometasone (Table 2), halometasone, triamcinolone acetonide, and methylprednisolone were ubiquitously applied in RCTs either as a primary comparator, an adjunct to another intervention, or part of a control group.

Calcipotriol is a vitamin D3 derivative approved for psoriasis and used in many inflammatory dermatologic conditions [220]. Vitamin D3 is also considered to have immunomodulating properties. For example, an in vitro analysis of peripheral blood mononuclear cells (PBMCs) showed that vitamin D3 represses inflammation [200]. Vitamin D3 promotes differentiation and proliferation of keratinocytes, thus promoting a strong epidermis [221]. Furthermore, a meta-analysis showed that in 1,840 patients with vitiligo, serum levels of vitamin D were lower in vitiligo patients compared to controls (p < 0.00001) [201]. Topical calcipotriol was used in six RCTs [16,19,100,101,105,143]. When combined with NB-UVB, the addition of calcipotriol was not superior to NB-UVB control [100,105]. Calcipotriol was used as a combination therapy in two studies but not statistically evaluated [16,101], and resulted in variable effects as a monotherapy: non-significant change in VASI when evaluated by one clinical trial, while significant in another (p=0.011) (Table 2) [19,143]. 

VITILSI® is a gel containing vegetable extracts from Coleus forskohlii root and Cassia alata leaf that have been reported to stimulate melanogenesis, downregulate the JAK/STAT pathway, and activate melanoblasts [97,222,223]. An RCT comparing VITILSI® gel alone and in combination with Bioskin® NB-UVB showed a slightly higher repigmentation rate in the combination group, though without statistical analysis [97].

Studies of superoxide dismutase in vitiligo cite the role of oxidative stress and inhibited function of catalase in the immunopathogenesis of vitiligo, with the idea that replacement of this peroxisomal enzyme with a pseudocatalase or superoxide dismutase will restore melanogenesis [224,225]. Topical application of a pseudocatalase/superoxide dismutase gel alongside tacrolimus ointment did not provide additional therapeutic benefit compared to tacrolimus alone [145]. An oral formulation of superoxide dismutase in combination with NB-UVB also failed to demonstrate significantly increased repigmentation versus placebo with NB-UVB [88].

The 'antioxidant cream' studied by Leone in 2015 included the following active ingredients: folic acid, phenylalanine, sitosterol, and phyto-sirtuin [20]. Folic acid reduces oxidative stress by activating the Nrf2 pathway and suppressing HMGB1, an inflammatory protein released during oxidative stress [226]. Phenylalanine, a precursor of tyrosine, contributes to melanin synthesis, and thus supplementation could improve synthesis in a deficient patient. Sitosterol and phyto-sirtuin are not well studied as treatments of vitiligo, and their mechanism of action is poorly understood. The mean repigmentation score was statistically significant between treatment and placebo cream (p < 0.05) [20].

Topical methotrexate has also been tested for vitiligo. One RCT comparing a combination of microneedling followed by methotrexate 1% gel demonstrated statistically better improvement in VASI versus microneedling followed by tacrolimus ointment at the 12-week follow-up (p = 0.009), though the data were not significantly different at the final endpoint (36 weeks) [125].

Topical 5-FU was used as a component of a control group [52,54,118], and as combination therapy [44,126-128]. Topical 5-FU monotherapy was minimally effective in one study: Abdelwahab et al. reported 0% to 5% repigmentation [52]. However, it is useful as a combination therapy with microneedling or Er:YAG laser [44,126-128]. 

Turmeric components turmeronol A and turmeronol B reduce NF-kappaB signaling in vitro [227]. A single trial found that turmeric cream significantly reduced VASI when compared to placebo cream (p < 0.001) (Table 2) [138]. Vitilinex Herbal Bio-Actives® is a combination of oil extracts known to have antioxidant properties (black cumin seed oil, black pepper, psoralea coryfolia, thymine oil, myrrh, and neroli) delivered in a lotion vehicle. The combination of Vitilinex® and NB-UVB was superior to either treatment alone, though statistical details were not provided [106]. Piperine is an alkaloid found in black pepper. While the mechanism of piperine in vitiligo has not been fully explored, it was found to be anti-inflammatory for psoriasis in a multiscale interactome protein analysis [228]. One trial investigated topical piperine in combination with NB-UVB and discovered greater mean pigmentation compared to placebo with NB-UVB (p = 0.005) [109].

Bergamot oil nanospanlastics employ nanomedicine for the targeted delivery of bergamot oil. There is some evidence to suggest that bergamot oil is anti-inflammatory. For example, oral administration of bergamot juice in rats with peritonitis led to decreased myeloperoxidase activity, decreased pro-inflammatory cytokines, and decreased activation of NF-KappaB [229]. In combination with NB-UVB, bergamot oil nanospanlastics performed better than NB-UVB alone (p < 0.05) [108].

Placental extract has been shown in vitro to have variable effects on melanogenesis, and the mechanism by which this occurs has not been well described [230]. Placental extract and mometasone furoate combination therapy were tested against tacrolimus and mometasone furoate combination therapy, and placental extract was not superior to tacrolimus [154].

Vitiskin® hydrogel (Isispharma) consists of a combination of superoxide dismutase, copper, zinc, vitamin B12, and vitamin B5. Zinc is considered to have anti-inflammatory properties via decreasing NF-KappaB activation [231]. Additionally, zinc and copper are necessary for melanogenesis. A meta-analysis of vitiligo patients found decreased serum zinc and copper, which could provide additional rationale for considering the two as adjuvant therapies in vitiligo [232]. In terms of vitamin B12, a systematic review conducted in 2019 found that vitiligo patients had higher serum homocysteine levels and lower vitamin B12 levels [233]. However, this is contrary to other evidence that vitamin B12 deficiency is associated with hyperpigmentation, and it is used in some topical formulations for bleaching [234]. There is also low evidence to suggest that vitamin B12 is associated with oxidative stress [235]. Limited data on the immunomodulatory effects of vitamin B5 suggest that it is pro-inflammatory, though a true association with inflammation in vitiligo is unknown [236]. Vitiskin® hydrogel was applied in combination with an excimer laser and resulted in significantly better repigmentation versus the excimer alone (p < 0.001) [23].

Prostaglandin F2-alpha analog (latanoprost) eyedrops commonly cause hyperpigmentation of the iris and periocular skin; thus, it was hypothesized that they may be effective in treating vitiligo. Their mechanism of action in vitiligo is not well described but is suggested to stimulate melanogenesis and melanosome transfer through the downstream pathway of alpha-MSH [237,238]. Topical PGF2α analog RCTs included in this review were numerous and focused on latanoprost [76,102,119,151] and bimatoprost [46,99,147,149]. Prostaglandins were generally effective as monotherapy and combination therapy.

The JAK inhibitors, as discussed in the systemic section, have also been tested topically. Topical application of ruxolitinib was studied by Rosmarin et al. in 2020 (phase II) and 2022 (phase III) and was successful as monotherapy in comparison to vehicle control (p < 0.001) (Table 2) [139,140]. 

Photocil® is a topical band-pass filter cream that selectively delivers NB-UVB when exposed to sunlight. It was created as a cost- and time-effective alternative to NB-UVB therapy. Photocil® was successful as monotherapy in comparison to placebo control (p < 0.0001) [146].

Tetracycline antibiotics have been shown to reduce the pro-inflammatory cytokines TNF-α and IL-1-β, inhibit metalloproteinases, and scavenge reactive oxygen species, thereby addressing several of the inflammatory processes that occur in vitiligo [239]. However, tetracycline ointment in combination with NB-UVB did not show a statistically significant improvement versus a placebo control (Table 2) [96]. 

Ethyl vanillate acts as an antioxidant by scavenging hydrogen peroxide. It has only been studied once in an RCT by Namazi et al. in 2015 [98]. Topical ethyl vanillate cream resulted in a significant reduction in VASI when combined with NB-UVB and resulted in significantly better repigmentation versus control (Table 2) (p = 0.005). 

Trichloroacetic acid (TCA) is a chemical peel that is hypothesized to induce repigmentation through controlled epidermal injury and wound healing, melanogenesis, and post-inflammatory hyperpigmentation [240]. Topical TCA in combination with microneedling resulted in greater repigmentation rates versus microneedling with pimecrolimus and microneedling with 5-FU (p = 0.0255) [126].

Basic fibroblast growth factor (bFGF) was suggested as a therapeutic target for vitiligo in 2013, on the basis that vitiligo patients have decreased mRNA expression of bFGF and that loss of bFGF has been associated with pigment loss [241]. A topical decapeptide solution derived from bFGF demonstrated significantly greater repigmentation rates versus the control tacrolimus group (p < 0.05) [153].

Cold atmospheric plasma (CAP)-activated hydrogel produces low-level reactive oxygen species (ROS). While ROS are typically considered part of the etiopathogenesis of vitiligo, some researchers have found that in low quantities, ROS regulate T-cell function [242]. The CAP treatment has also been shown to reduce lymphocyte function-associated antigen 1 and ICAM1 in other inflammatory models, such as the psoriasis mouse model [243]. Monotherapy resulted in a statistically significant reduction in VASI (p = 0.0012), though raw data were not reported, and statistical comparison to the control was not provided (Table 2) [144].

Other Therapies

Low-intensity pulsed ultrasound (LIPUS) uses acoustic output to heat or ablate tissue. It has recently gained popularity in aesthetics for its potential to promote skin elasticity, reduce cellulite, and contour the body [244]. It has been shown to increase the proliferation of fibroblasts, protein synthesis, and aid in melanoblast migration. It was successful with concomitant therapies of NB-UVB or topicals (corticosteroids and calcineurin inhibitors) in truncal vitiligo (p = 0.046) [58].

Summary of RCTs

From 2013 to 2023, most clinical trials focused on topical therapy as a treatment for vitiligo (20 studies), closely followed by phototherapy and systemic combination therapy (18 studies), phototherapy and topical combination therapy (16 studies), and surgical and topical combination therapy (12 studies). Of the 151 studies extracted, 106 RCTs investigated combination therapy.

Studies including the VASI measure of repigmentation as a primary outcome

Of the 151 RCTs, 36 studies employed VASI as a primary outcome measure (Table 5) [11,33,34,51,59,60,68,77,78,80-86,94-96,98,99,117-119,129-133,138-144]. Our focused qualitative analysis of studies using VASI as an endpoint identified 30 studies that could be grouped into three intervention categories: phototherapy and systemic (Table 3), topical or topical combination (Table 2), and systemic monotherapy (Table 4). Of these 30 studies, 12 had statistically significant results compared to the control [77,84-86,98,99,117,138-140,142,143]. Of these 12, eight utilized combination therapy (Tables 2-4) [77,84-86,98,99,117,142]. This is consistent with the existing literature, which shows that combination therapy is superior to monotherapy. 

**Table 5:** Studies including the VASI measure of repigmentation as a primary outcome Each RCT was categorized as described in the text. Combination therapies are denoted by a '+' between each category. The rows list intervention categories, along with references to the RCTs that investigated the intervention method. The 'Count' column shows the 36 RCTs which recorded VASI as an outcome measure [11,33,34,51,59,60,68,77,78,80-86,94-96,98,99,117-119,129-133,138-144]. The RCTs featured in Tables 2-4 (n=30) are specified under the 'intervention category' column in parentheses. VASI: Vitiligo area scoring index, RCTs: Randomized controlled trials

Intervention category	References	Count
Surgical + topical (Table 2)	[117-119]	3
Excimer + phototherapy	[11]	1
Laser	[33]	1
Laser + phototherapy	[34]	1
Laser + topical (Table 2)	[51]	1
Phototherapy	[59,60]	2
Phototherapy + surgical	[68]	1
Phototherapy + systemic (Table 3)	[77,78,80-86,94]	10
Phototherapy + topical (Table 2)	[95,96,98,99]	4
Systemic (Table 4)	[129-133]	5
Topical (Table 2)	[138-144]	7
Total		36

**Figure 4:**
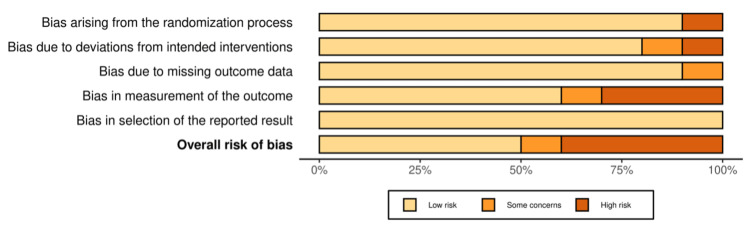
Risk of bias of phototherapy and systemic RCTs Risk of bias of the RCTs (n=10) was assessed using the revised Cochrane risk of bias (RoB 2) tool for randomized trials [158]. The risk of bias plot was made using the robvis tool. Overall risk of bias demonstrated low risk in 50% of studies, some concerns in 10% of studies, and high risk in 40% of studies [77,78,80-86,94]. RCTs: Randomized controlled trials

Ten RCTs investigated combination phototherapy and systemic therapy, using VASI as an endpoint (Table 3). Interventions included psoralen (2), afamelanotide, apremilast, minocycline, vitamin D, atorvastatin, vitamins A and E, prednisone, and silybum marianum [77,78,80-86,94]. Nine of 10 studies showed statistically significant reduction in VASI after treatment compared to baseline, further supporting the efficacy of phototherapy [77,80-86,94]. Treated versus control subjects showed statistically improved VASI after the following interventions were added to phototherapy: psoralen, minipulsed prednisone, afamelanotide, and vitamin A and E [77,84-86]. Overall risk of bias demonstrated low risk in 50% of studies, some concerns in 10% of studies, and high risk in 40% of studies (Figure 4). 

**Figure 5:**
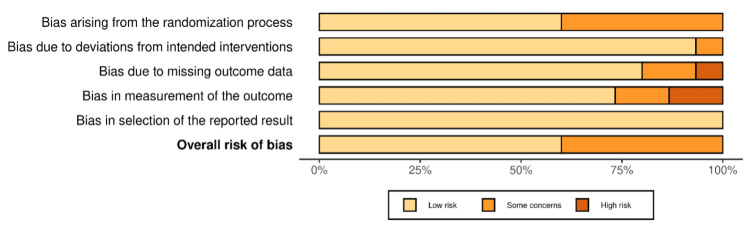
Risk of bias of topical and topical combination therapy RCTs Risk of bias of the RCTs (n=15) was assessed using the revised Cochrane risk of bias (RoB 2) tool for randomized trials [158]. The risk of bias plot was made using the robvis tool. Overall risk of bias demonstrated low risk in 60% of studies, and some concerns in 40% of studies. RCTs: Randomized controlled trials

Fifteen RCTs examined topical or topical combination treatments (Table 2). Treatment duration ranged from three to 12 months. Studies in this category examined microdermabrasion, CO2 laser (2), suction blister alone versus dermabrasion plus 5-FU, microneedling, psoralen, turmeric, tetracycline, ethyl vanillate cream, tacrolimus, bimatoprost with NB-UVB, mometasone furoate, betamethasone with calcipotriol, ruxolitinib, and cold atmospheric plasma-activated hydrogel [51,95,96,98,99,117-119,138-144]. Eight of 15 studies showed statistically significant reduction in VASI after treatment compared to baseline [98,99,117,138-140,142,143]. Of those eight studies, four employed combination therapy, further supporting the efficacy of combination therapy [98,99,117,142]. Treated versus control subjects showed statistically improved VASI after the following interventions: microdermabrasion with tacrolimus, turmeric monotherapy, ethyl vanillate cream with NB-UVB, bimatoprost with and without NB-UVB, tacrolimus with mometasone furoate, ruxolitinib (in two studies), and betamethasone dipropionate with calcipotriol when compared to calcipotriol alone [98,99,117,138-140,142,143]. Overall risk of bias demonstrated low risk in 60% of studies, and some concerns in 40% of studies (Figure 5). 

**Figure 6:**
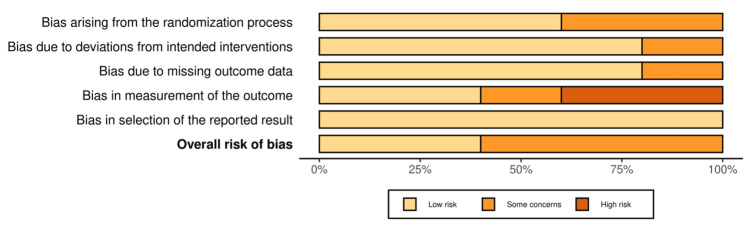
Risk of bias of systemic therapy RCTs Risk of bias of the RCTs (n=5) was assessed using the revised Cochrane risk of bias tool (RoB 2) for randomized trials [158]. The risk of bias plot was made using the robvis tool. Overall risk of bias demonstrated low risk in 40% of studies, and some concerns in 60% of studies [129-133]. RCTs: Randomized controlled trials

Five trials reported outcomes on systemic treatments without other prescribed interventions (Table 4); however, none reported statistically significant differences [129-133]. A placebo-controlled study on Ginkgo biloba supplementation in 50 patients found a 4% reduction in VASI [129]. A small study on simvastatin showed a 26% reduction in VASI over six months [133]. Three trials compared dexamethasone OMP with cyclosporine, minocycline, and methotrexate, respectively, and demonstrated differential results with regard to VASI [130-132]. Overall risk of bias demonstrated low risk in 40% of studies and some concerns in 60% of studies (Figure 6). 

Discussion

The last systematic review to summarize the published RCTs on vitiligo therapies updated prior versions of a Cochrane review for a total of 96 RCTs up to 2013 [5,245,246]. Our review of 151 RCTs published from 2013 to 2023 aimed to encapsulate changes in the literature for dermatologists and others managing the care of patients with vitiligo. Other systematic reviews for vitiligo treatment in the literature within the last five years have focused on specific treatment modalities, including topicals, phototherapy, lasers, systemics, surgical therapies, alternative therapies, and combinations thereof [164,178,247-251]. For example, a systematic review and meta-analysis of surgical vitiligo therapies found that combining minimally invasive surgical techniques such as microneedling or lasers with NB-UVB produced favorable repigmentation results while mitigating adverse effects [178]. Another systematic review of autologous transplantation (referred to by the authors as regenerative medicine) found that it has success alone and is often improved by combination with other vitiligo treatments [252]. 

In recent years, JAK inhibitors have risen to the forefront of dermatologic research and have also demonstrated success in vitiligo in the TRuE-V1 and TRuE-V2 trials, culminating in ruxolitinib cream being the first FDA-approved treatment for refractory nonsegmental vitiligo [140]. Again, combination regimens are key in treating vitiligo, as a review found that 95% of patients on an oral JAK inhibitor with phototherapy experienced a complete or partial response [253,254]. Oral JAK inhibitors show promise but are generally used with great caution due to the five boxed warnings for serious adverse events, though recent studies investigating their safety in dermatologic inflammatory conditions have not identified a significantly increased risk of feared complications [255,256]. A thorough discussion of risks and benefits is necessary for any treatment.

A limitation of this systematic review is that it was pooled from a highly heterogeneous population of RCTs, many of which measured outcomes of interest differently. The heterogeneity of studies included made meta-analysis unfeasible. This observation was confirmed by a study evaluating the implementation of the core outcome set for vitiligo, which found that between 2009 and 2021, 38 different outcomes were reported by RCTs, and only 11 of 112 RCTs reported all three primary outcome measures [257]. The authors concluded that efforts are needed to improve utilization of the core outcome set. We concur and recommend that future clinical studies adopt the e-Delphi consensus on a core outcome set for vitiligo RCTs to homogenize data and improve the ability to conduct meta-analyses [258]. While this review’s search was quite sensitive for RCTs, we acknowledge that many non-RCT studies of therapies in vitiligo were omitted; more robust clinical trials are necessary to provide the highest quality evidence for treatment. We would also like to emphasize that not all interventions studied between 2013 and 2023 were successful and viable treatment methods, and this systematic review was created to guide physicians through their search of the literature and inform discussion on the optimal course of treatment alongside each patient.

The search for new therapeutic targets in vitiligo can be informed by high-throughput analysis of vitiligo lesions. The continuing advancement of single-cell RNA sequencing (sc-RNA-seq) technology may also provide new insights into future directions of therapeutics. In a sample of eight patients (five non-segmental vitiligo and three control patients), PBMC analysis revealed changes in the following genes and pathways: regulatory T cells, natural killer cells, dendritic cells, B cells, monocytes, and neutrophils [259]. In a larger sample of 10 vitiligo patients and seven control patients, suction blister samples from lesional skin scRNA-seq analysis revealed that lesional samples were associated with macrophages, T cells, and melanocytes, and additional mouse studies from the same project revealed that CCR5 may have a role in regulatory T cell migration [260]. 

Materials and methods

Literature Search

Two reviewers (NMR and ZJ) searched the following databases: Clinicaltrials.gov, Cochrane Library, Embase, PubMed, Scopus, and Web of Science from October 2013 to October 2023. Any results for vitiligo treatment in human subjects published in English were included in the preliminary search. Search terms used to produce results were "vitiligo" and/or "treatment" or "intervention." Two reviewers (NMR and ZJ) independently reviewed titles and abstracts for eligibility using the Covidence systematic review management program (Cochrane, London, GBR) and included all relevant texts in the full-text review. Full texts were screened by two reviewers (NMR and ZJ), and all applicable full texts underwent extraction. Inconsistencies were resolved by consensus with a third reviewer (DJL). 

Inclusion and Exclusion Criteria

Our inclusion criteria were English- or Spanish-language RCTs of non-segmental vitiligo patients that measured any outcome of interest. We excluded studies that did not differentiate segmental versus non-segmental vitiligo, studies that were not randomized, studies that did not have control groups, studies that did not implement a treatment for vitiligo, gray literature, unpublished work, non-human studies, duplicate studies, and trials that were not randomized and controlled. Some homeopathic therapy studies were excluded from this review due to inadequate control (two homeopathic therapies tested and compared against each other). However, when homeopathic treatments were accompanied by adequate control, they were included in the systematic review. 

Data Extraction

Mean age, gender ratio, country, sample size, body site, and duration of treatment were extracted from the records. Interventions were categorized according to type of treatment: excimer, laser, phototherapy, topical, systemic, surgery, intralesional, or other. When not reported by the study, the change in VASI was calculated by determining the difference in means, as well as combining the SD of the two independent variables. Data extraction focused on VASI score outcomes listed for total body sites, as this information was available for all studies that collected VASI scores. We used the Revised Cochrane risk-of-bias (RoB 2) tool for randomized trials to assess the risk of bias of evidence included in the review. Two reviewers (NMR and ZJ) independently evaluated the overall quality of the evidence. Risk-of-bias plots were made using the robvis tool [261]. 

Outcomes

The primary and secondary outcomes of interest were determined using a core outcome set for vitiligo trials established in 2015 via an international e-Delphi consensus [258]. The 2015 guidelines include "repigmentation, side effects and harms of treatment… maintenance of gained repigmentation” as primary outcomes of interest [258]. Though these guidelines have been identified as the gold standard in measuring core outcomes, we have observed little buy-in amongst researchers in using these specific criteria since their release in 2015. Therefore, in addition to the recommended primary and secondary outcomes, we also recorded any other measurements relating to response to vitiligo treatment. Primary outcomes of interest included repigmentation percentages, quartiles or other categorical quantiles, VASI, physician’s global assessment, vitiligo extent score, self-assessment for vitiligo extent score, depigmentation degree, Vitiligo European Task Force, side effects or adverse events; descriptive statistics as reported by trials; and maintenance of gained repigmentation based on the above pigmentation indices and the percentages of categorical yes/no. Secondary outcomes were the 2015 e-Delphi consensus, which also listed the following as additional outcomes: “cosmetic acceptability of results, quality of life, cessation of spreading of vitiligo, and tolerability/burden of treatment” [258]. Thus, we included the following measures featured in the studies: cosmetic acceptability (Vitiligo Noticeability Questionnaire); quality of life (Dermatology Life Quality Index, Vitiligo Impact Patient Scale, Skindex-29, and Vitiligo-Specific Quality-of-Life Instrument (VitiQoL)); cessation of disease activity (VIDA, Vitiligo European Task Force Vitiligo Extent Score, and VASI); and tolerability and burden of treatment as reported by trials.

## Conclusions

This systematic review sought to provide a snapshot of the past 10 years of vitiligo RCTs as a brief but informative reference for dermatology providers. In sum, we found that 106 of 151 RCTs from 2013 to 2023 studied combination therapy, and combination therapy is superior to monotherapy. Overall, it is important to recognize that vitiligo has a multifactorial etiology, and thus combination therapies are the most appropriate for treatment. 
